# Supplementary figures and images for: Knockdown of microglial iron import gene, Slc11a2, worsens cognitive function and alters microglial transcriptional landscape in a sex-specific manner in the APP/PS1 model of Alzheimer’s disease
Source: J Neuroinflammation. 2024 Sep 27;21:238. doi: 10.1186/s12974-024-03238-w (PMC11438269; doi:10.1186/s12974-024-03238-w)

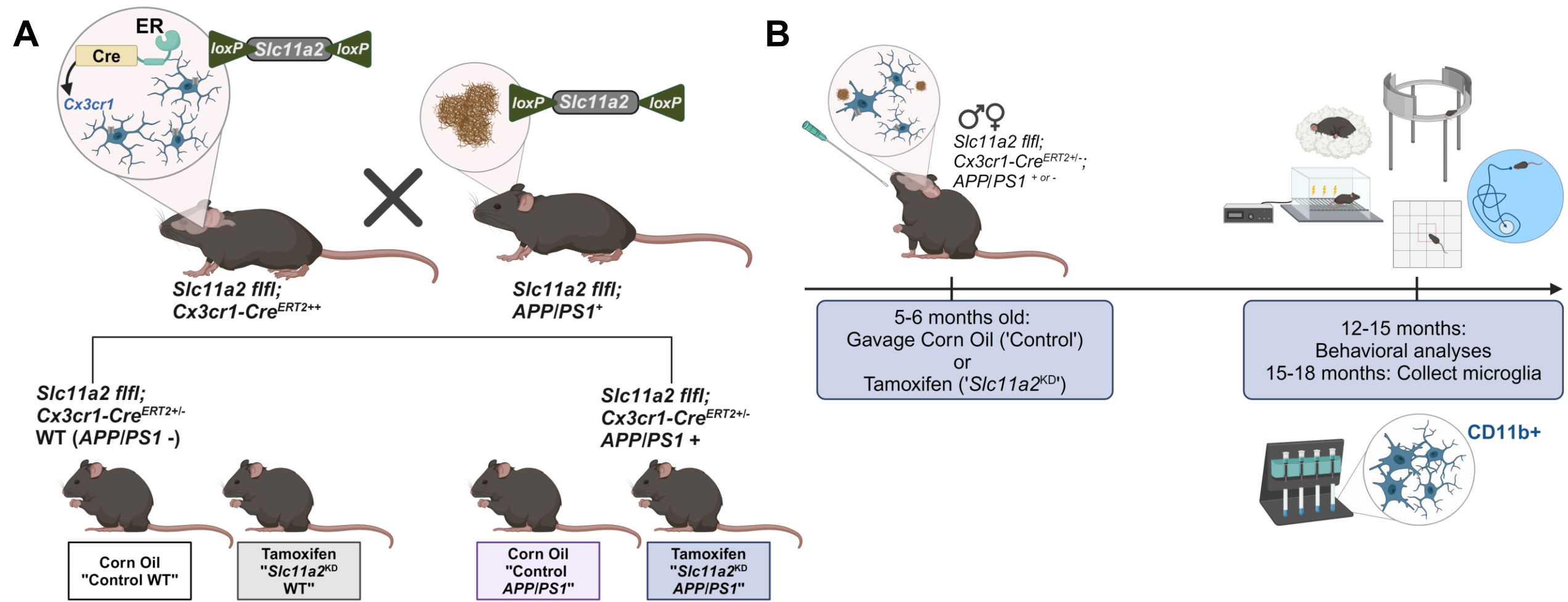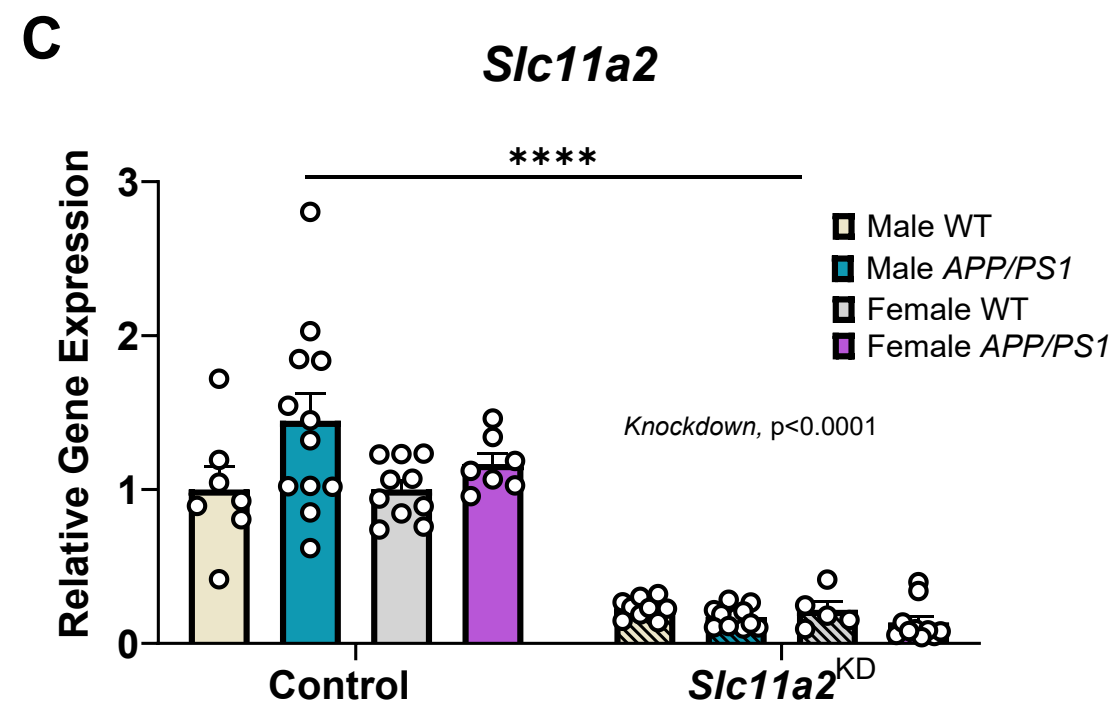

Supplement: Supplementary file 1 — Additional file 1. Experimental model and timeline diagram. A Schematic of mouse model generated in these studies. Slc11a2flfl;Cx3cr1Cre-ERT2+/+ mice were bred with Slc11a2flfl;APP/PS1+ hemizygotes to yield two resultant genotypes: Slc11a2flfl;Cx3cr1Cre-ERT2+/-;APP/PS1+ and Slc11a2flfl;Cx3cr1Cre-ERT2+/-;WT. Tamoxifen was administered to half the animals to induce knockdown of microglial Slc11a2, and corn oil was used as a control. This resulted in four experimental groups: Control WT, Slc11a2KD WT, Control APP/PS1+, and Slc11a2KD APP/PS1+. B Timeline of experiments. Tamoxifen gavage was used to induce Slc11a2 knockdown at 5-6 months of age, and behavioral analyses were conducted between 12-15 months of age. Tissue was collected when mice were 15-18 months of age. C CD11b+ microglial cells were isolated from whole brains, and confirmation of Slc11a2 knockdown was done via RT-qPCR using a primer targeting exons 7-8 in both sexes. Two-way ANOVA, ****p<0.0001 effect of knockdown. [file 12974_2024_3238_MOESM1_ESM.pdf]

**A**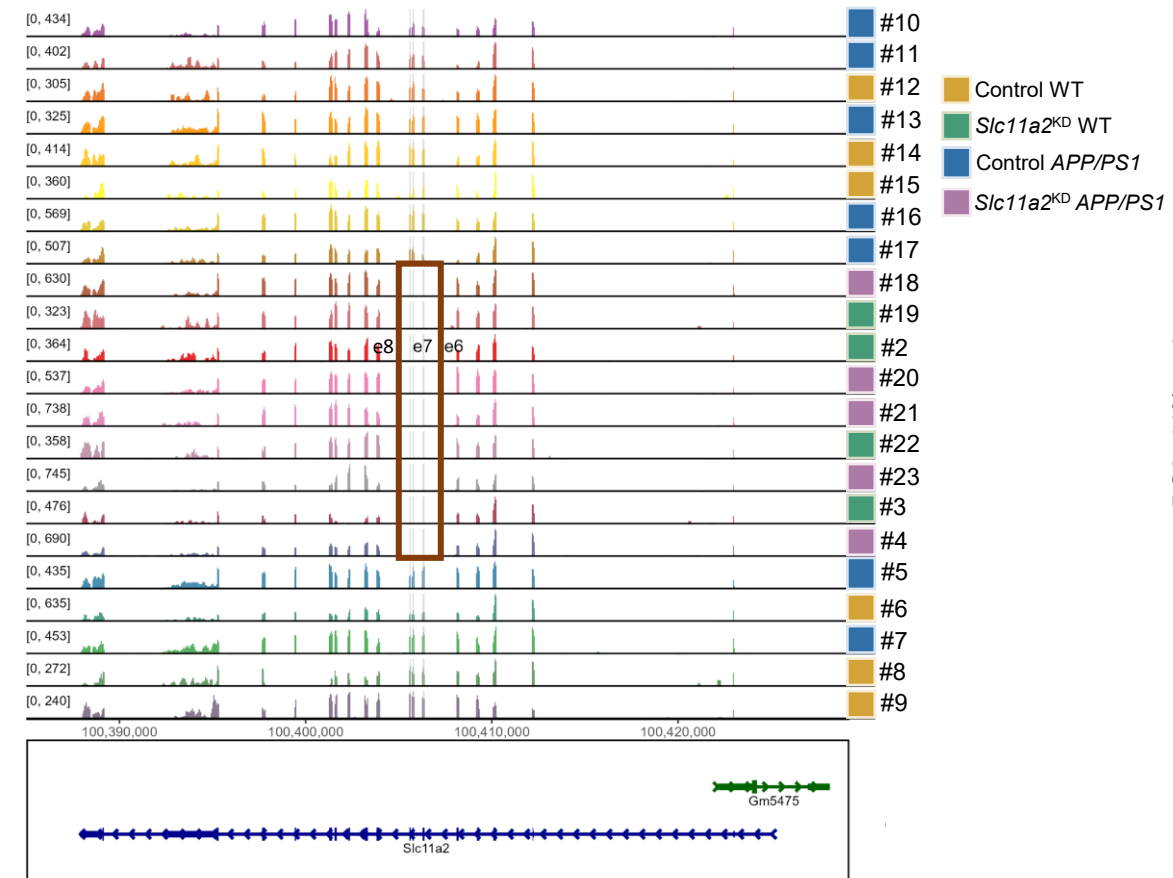**B**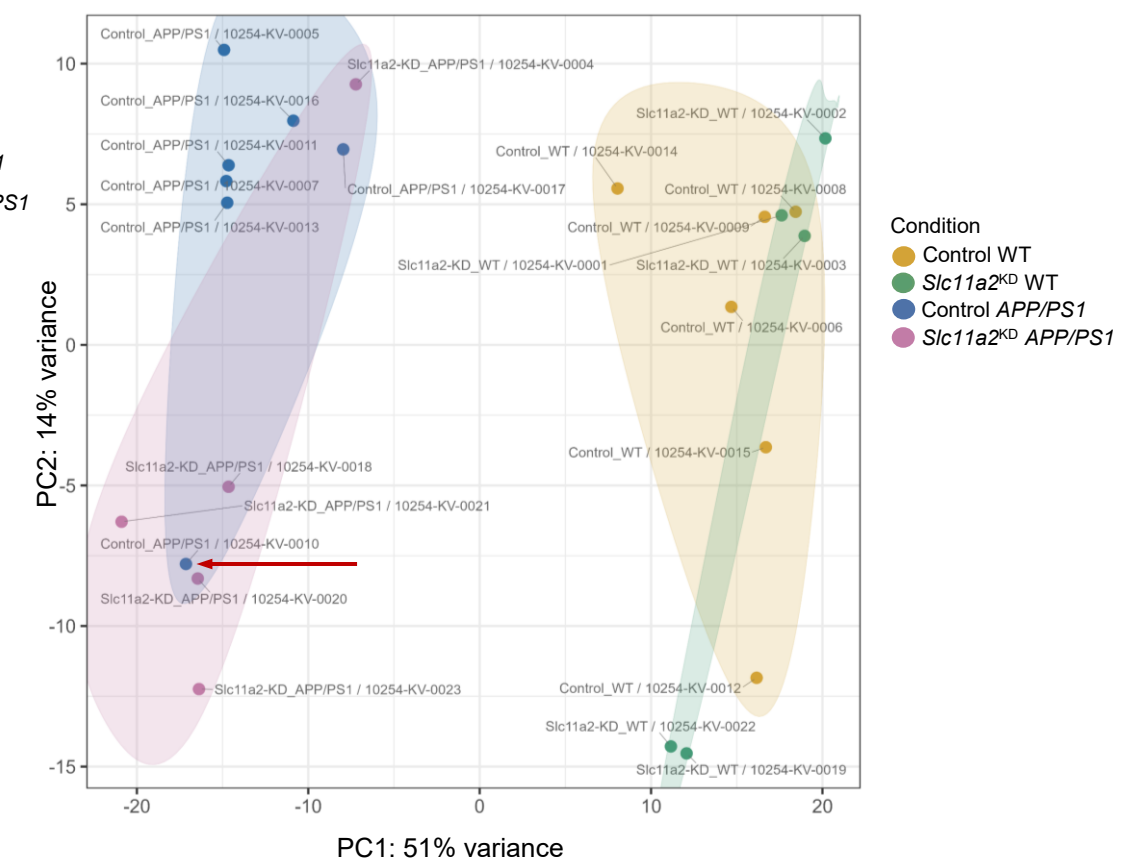**C****Outlier Diagnostics: Control\_ *APP/PS1***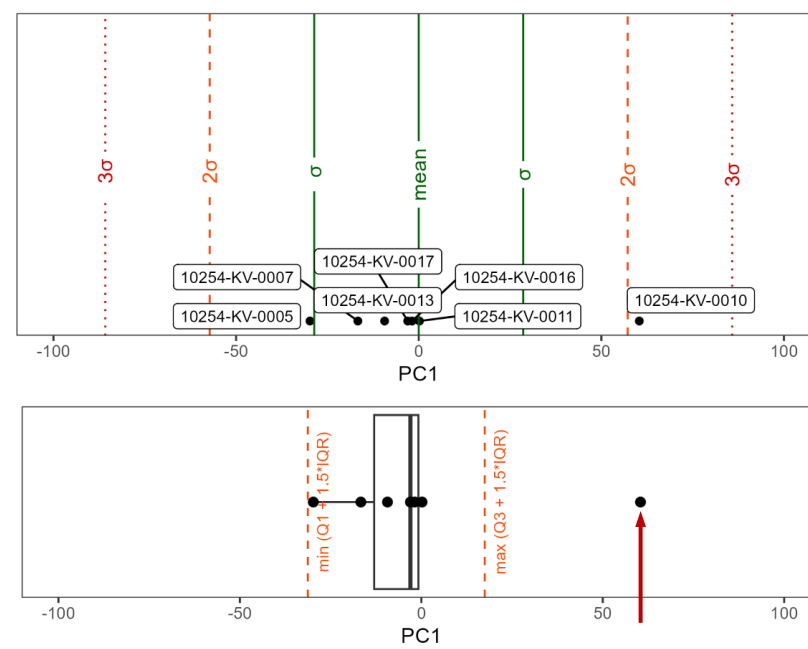

Supplement: Supplementary file 4 — Additional file 4. Knockdown confirmation and outlier analysis in female microglia from RNA-seq. A RNA-seq read coverage across annotated exons in the Slc11a2 gene using the package, ggcoverage v1.3.0. Slc11a2KD samples exhibited complete abrogation of reads between exons 6-8. Sample ID numbers shown on right of plot. B PCA plot showing all samples in RNA-seq analysis. The red arrow is pointing to sample #10 in control APP/PS1 group. C Outlier analysis of control APP/PS1 group, showing #10 as statistical outlier. Data represent 5-7 mice per group. [file 12974_2024_3238_MOESM4_ESM.pdf]

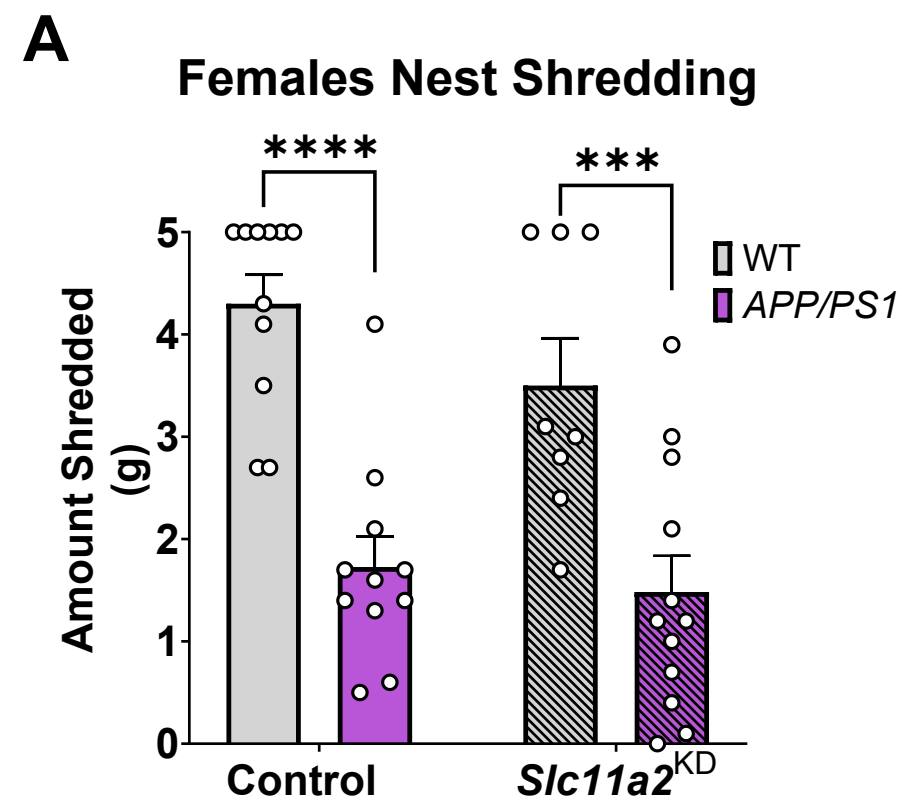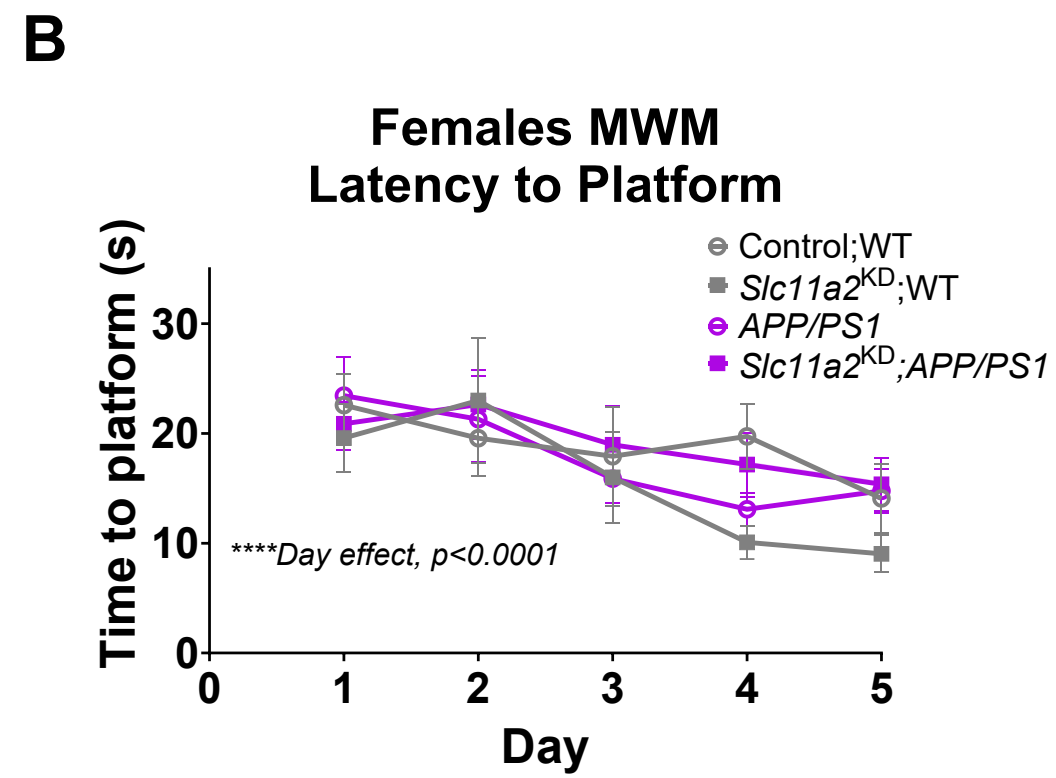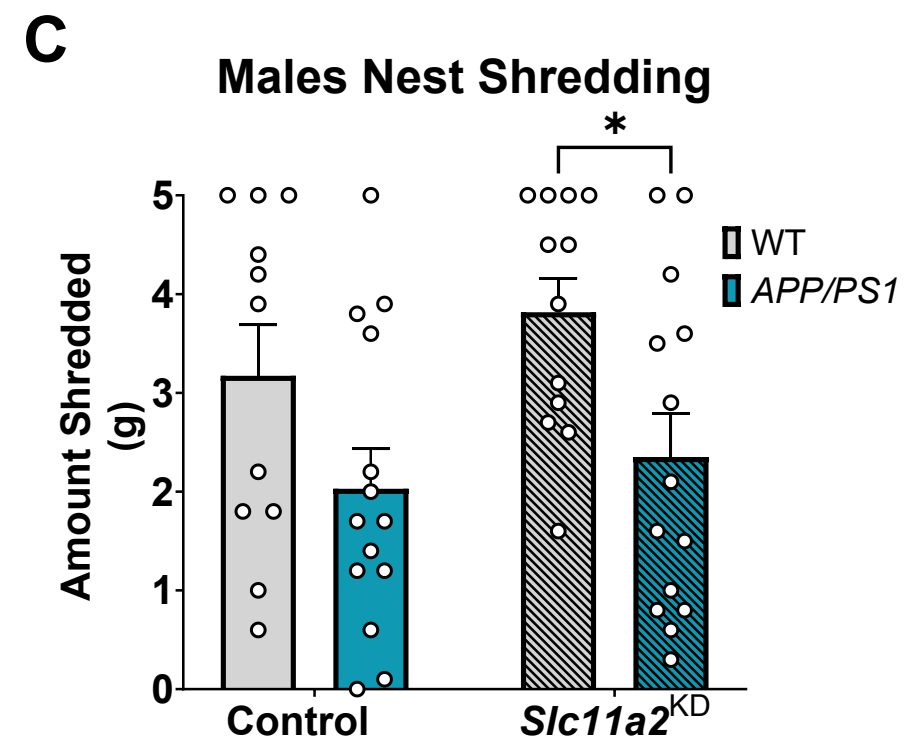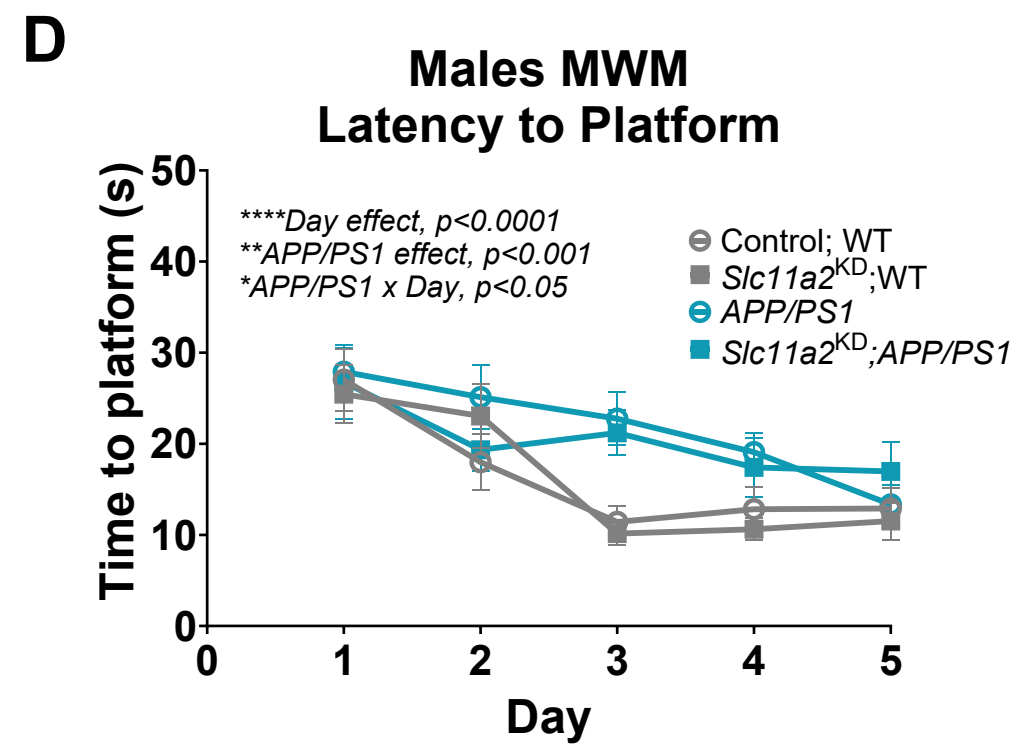

Supplement: Supplementary file 5 — Additional file 5. Slc11a2 knockdown had no additional effect on APP/PS1-associated deficits in nest building or Morris water maze latency. A and C Amount nestlet shredded after overnight nest building assay. Two-way ANOVA, *p<0.05, **p<0.01, ***p<0.001, ****p<0.0001. B and D Latencyto reach hidden platform of Morris water maze during training days. Four trials per day were averaged for one data point per animal, and these trials were repeated for five days. Three-way ANOVA, *p<0.05, **p<0.01, ****p<0.0001. Data represent the mean ± S.E.M. of 8-14 mice per group. [file 12974_2024_3238_MOESM5_ESM.pdf]

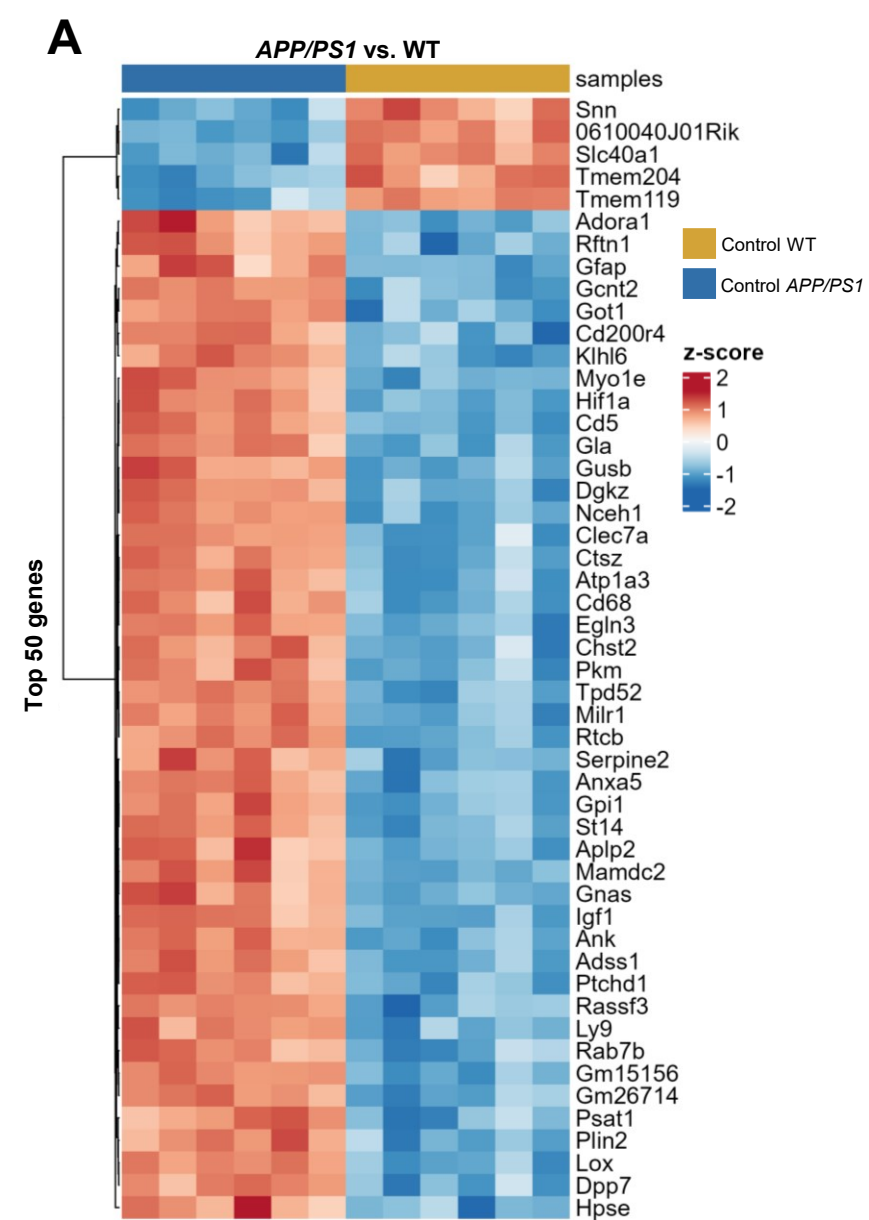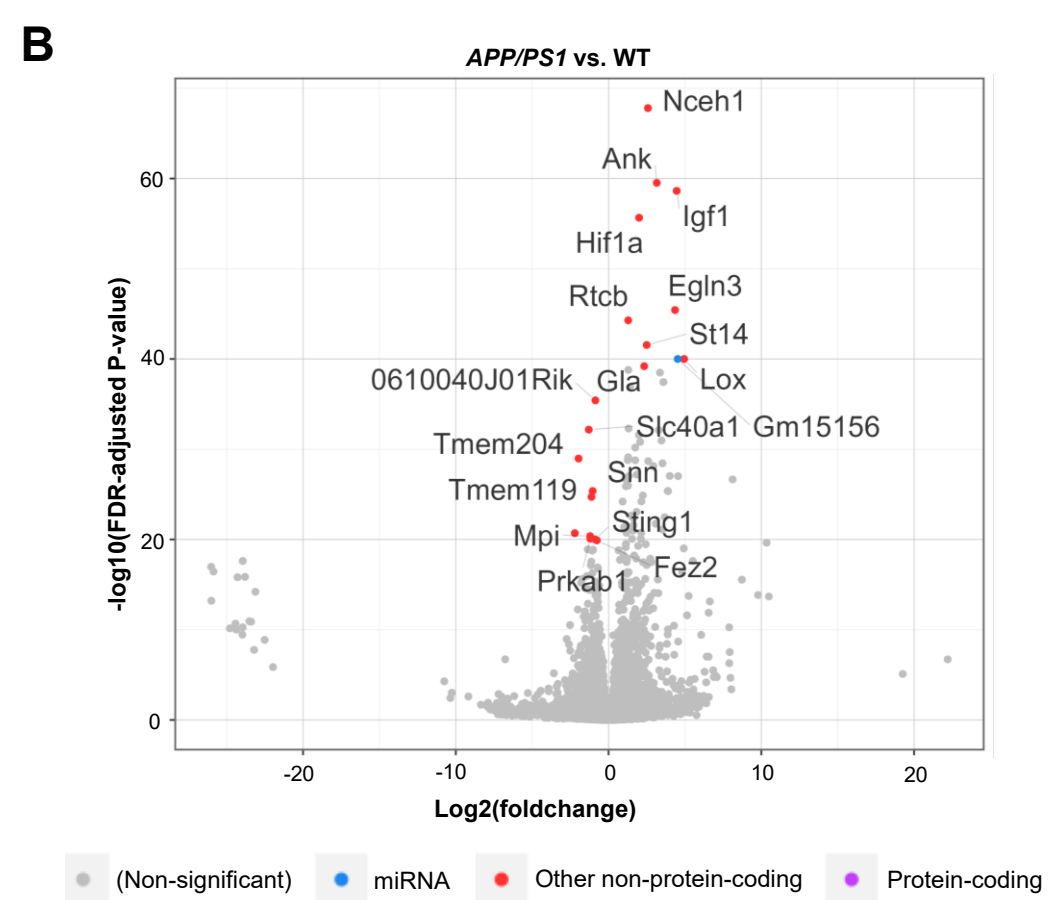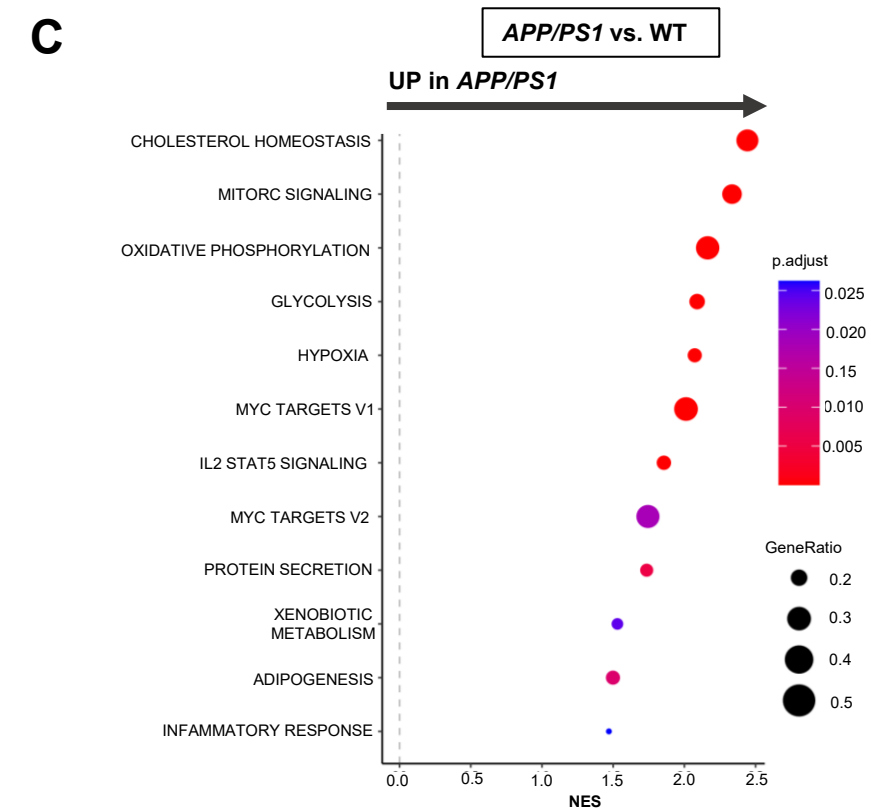

Supplement: Supplementary file 6 — Additional file 6. Hippocampal microglia from female APP/PS1 mice exhibit significant alterations in gene expression compared to WT. A Top 50 DEGS by adjusted p-value between APP/PS1 and WT microglia. Heat map shows upregulations in APP/PS1 cells in red and downregulations in blue. B Volcano plot showing genes differentially alteredin APP/PS1 compared to WT microglia. C GSEA of significantly altered hallmark gene sets in APP/PS1 cells compared to WT. Upregulated pathways in APP/PS1 microglia include those involved in cholesterol homeostasis, inflammatory signaling, and metabolic changes. Data represent 5-6 mice per group. [file 12974_2024_3238_MOESM6_ESM.pdf]

**A**

| Differentially expressed genes where <i>padj</i> < 0.05 |            |            |            |            |
|---------------------------------------------------------|------------|------------|------------|------------|
| Regulation                                              | Log2FC > 0 | Log2FC > 1 | Log2FC > 2 | Log2FC > 3 |
| down                                                    | 4          | 4          | 4          | 4          |
| up                                                      | 6          | 6          | 5          | 5          |

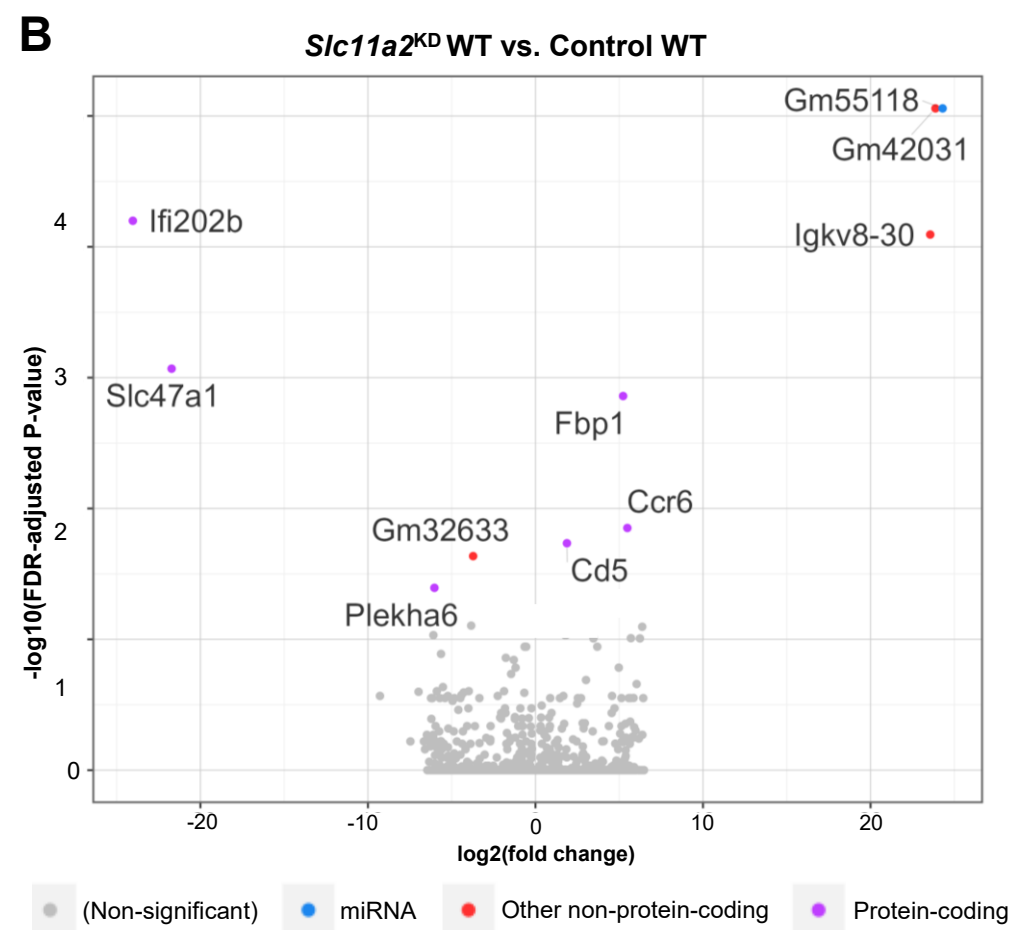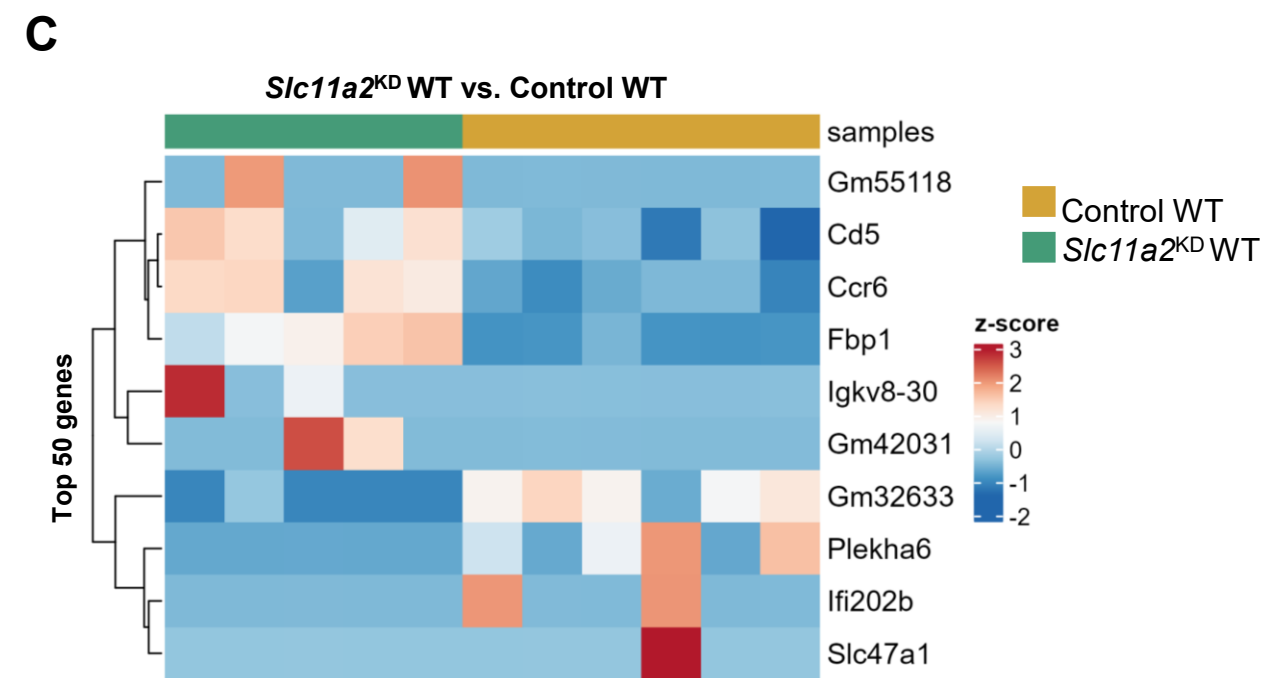

Supplement: Supplementary file 7 — Additional file 7. Slc11a2KD microglia from WTfemale mice exhibit minimal alterations in gene expression compared to controls. A Number of DEGS from RNA-seq analysis comparing control WT and Slc11a2KD WT groups. B Volcano plot showing genes differentially expressedin Slc11a2KD versus control WT microglia. C Heat map showing top DEGSin Slc11a2KD versus control WT microglia. Red = upregulated, blue = downregulated. Data represent 5-6 mice per group. [file 12974_2024_3238_MOESM7_ESM.pdf]

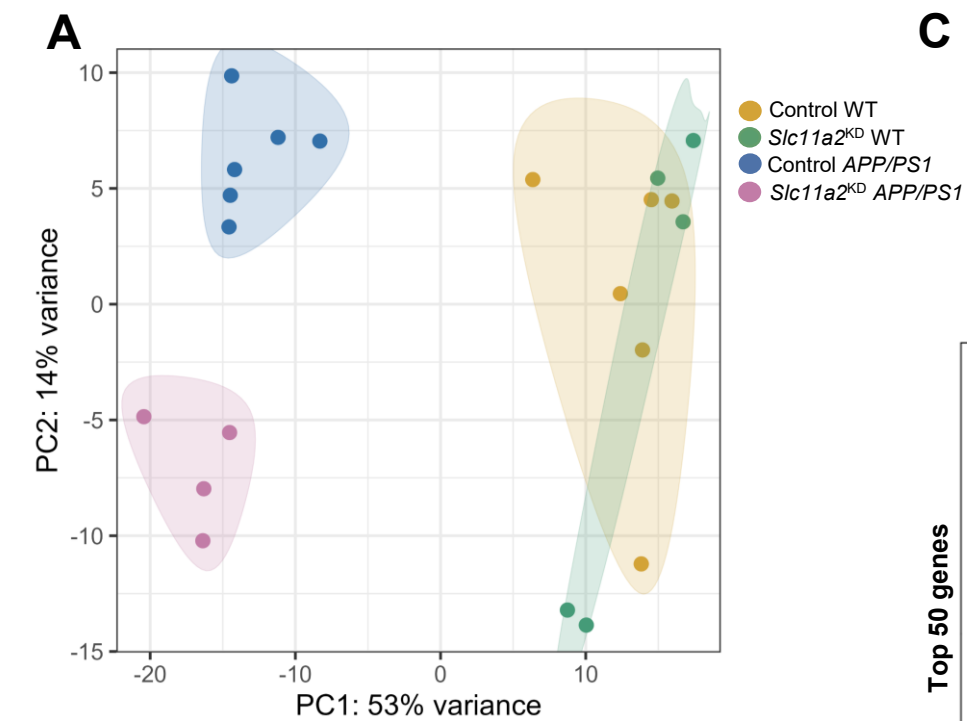

**B**

Differentially expressed genes where *padj* < 0.05

| Regulation | Log2FC > 0 | Log2FC > 1 | Log2FC > 2 | Log2FC > 3 |
|------------|------------|------------|------------|------------|
| down       | 2210       | 509        | 15         | 1          |
| up         | 2230       | 204        | 42         | 23         |

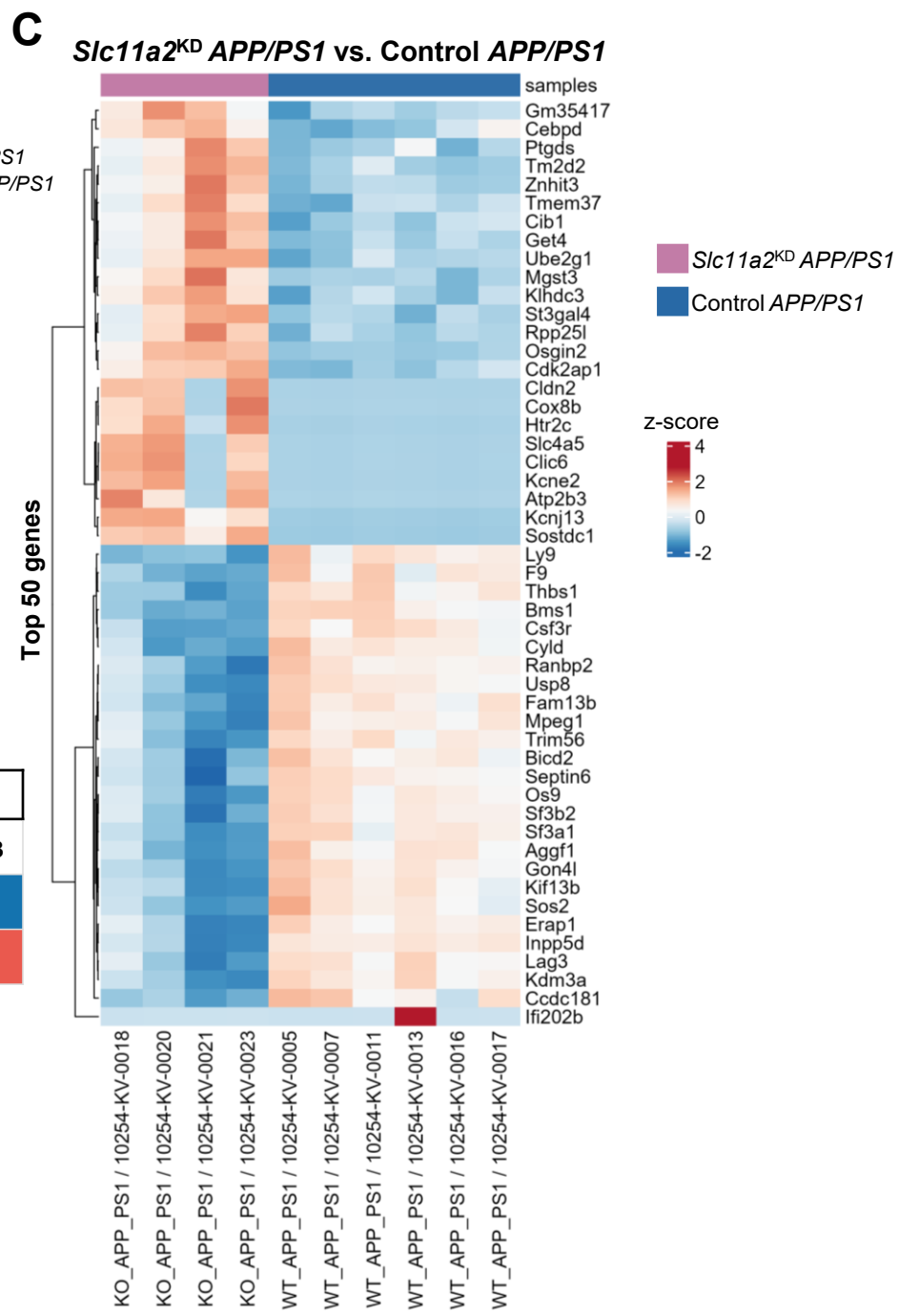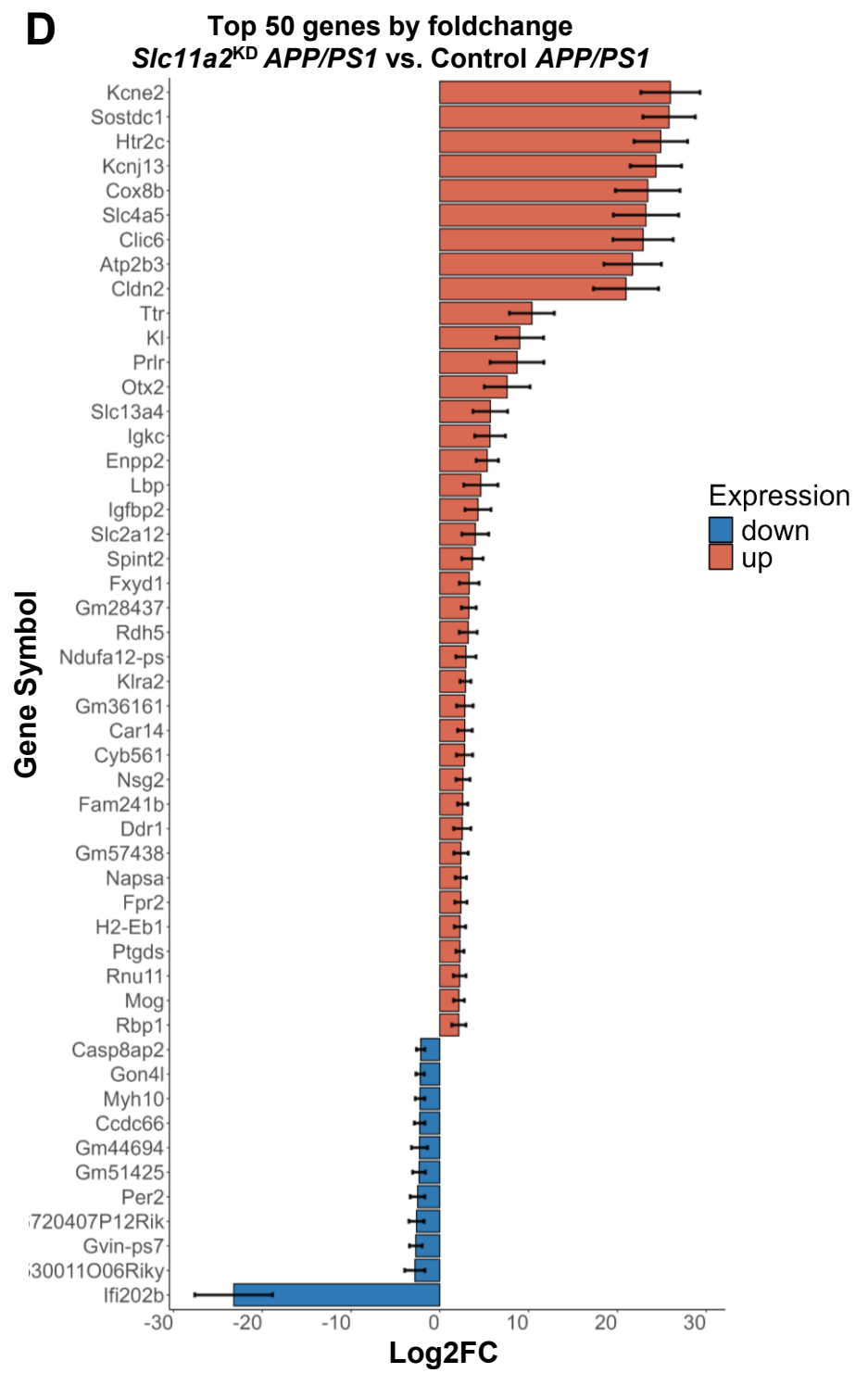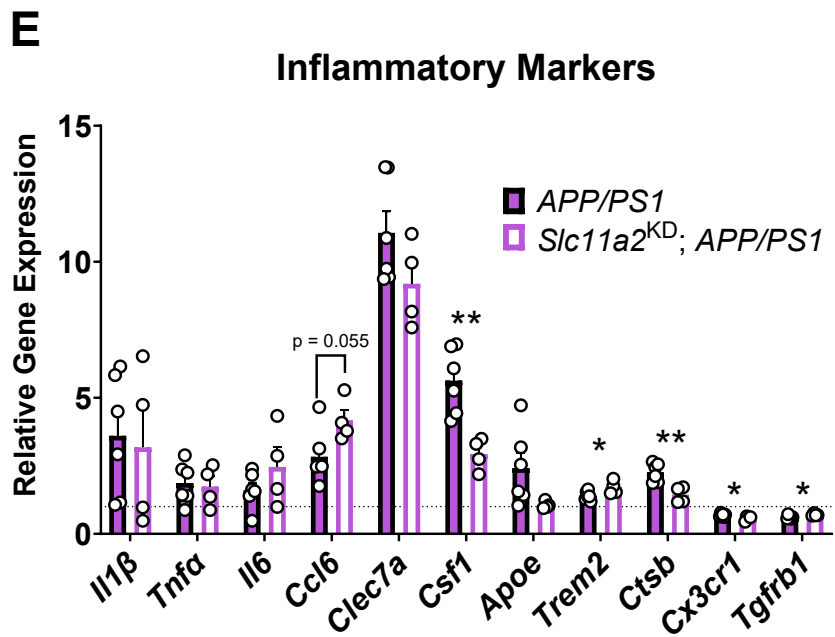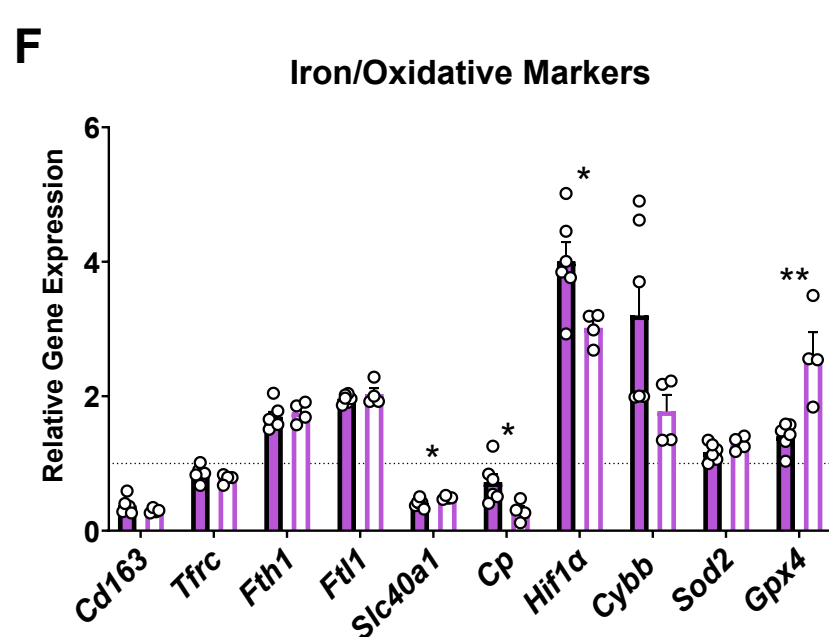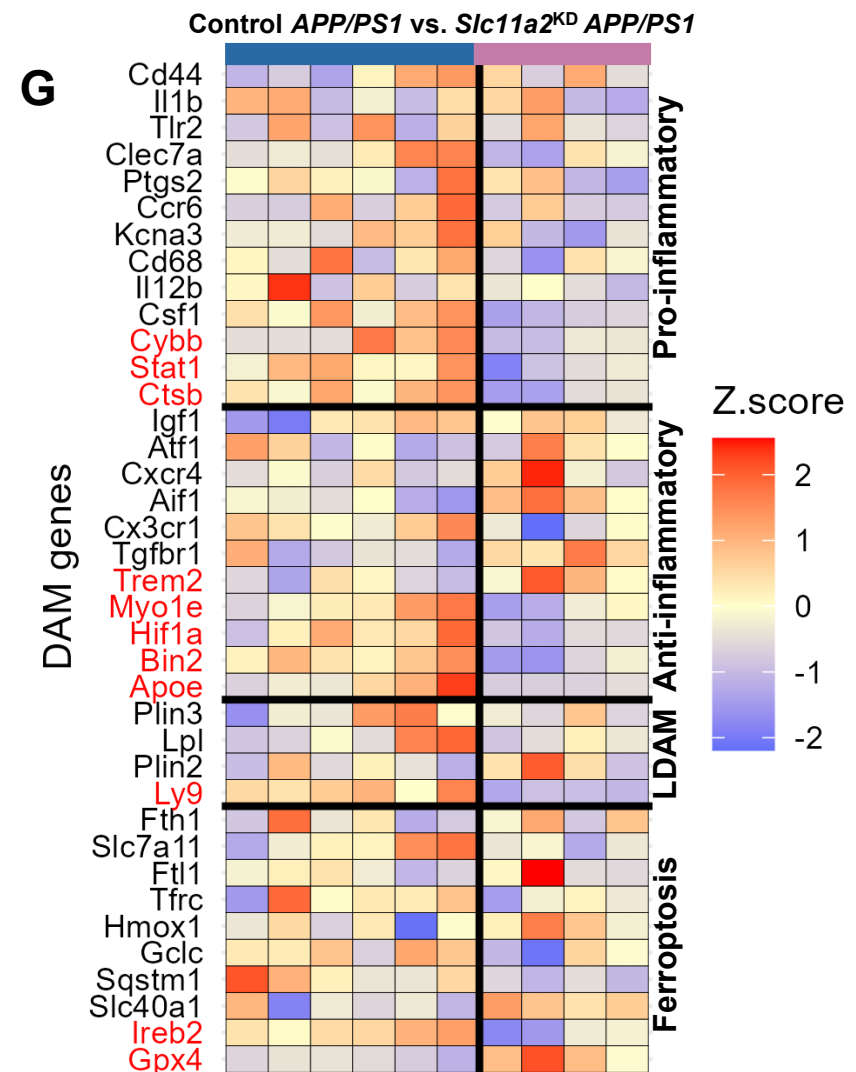

Supplement: Supplementary file 8 — Additional file 8. Removal of variable sample from Slc11a2KD APP/PS1 group in RNA-seq data reveals robust effects of Slc11a2KD on microglial gene expression in APP/PS1 female mice. A–D Data from RNA-seq analysis when sample #4 is removed. A PCA plot showing separation of group clusters. B Number of DEGs between Slc11a2KD and control APP/PS1 microglia. C Heat map showing top 50 DEGSbetween Slc11a2KD and control APP/PS1 microglia. Red = upregulated, blue = downregulated. D Top 50 DEGs by log fold-change between Slc11a2KD and control APP/PS1 microglia. E, F Targeted gene expression analysis from RNA-seq dataset showing changes in E inflammatory markers and F iron-related and oxidative stress markers from Slc11a2KD versus control APP/PS1 microglia. Gene expression is shown relative to control WT group set to 1. *p<0.05, **p<0.01 student’s t-test. G Gene markers representing subsets of DAMswere analyzed via RNA-seq between Control and Slc11a2KD APP/PS1 female microglia after removing sample #4. Genes highlighted in red are significantly different between groups, adjusted p-value < 0.05. Data represent mean ± S.E.M. of 4-6 mice per group. [file 12974_2024_3238_MOESM8_ESM.pdf]

**A**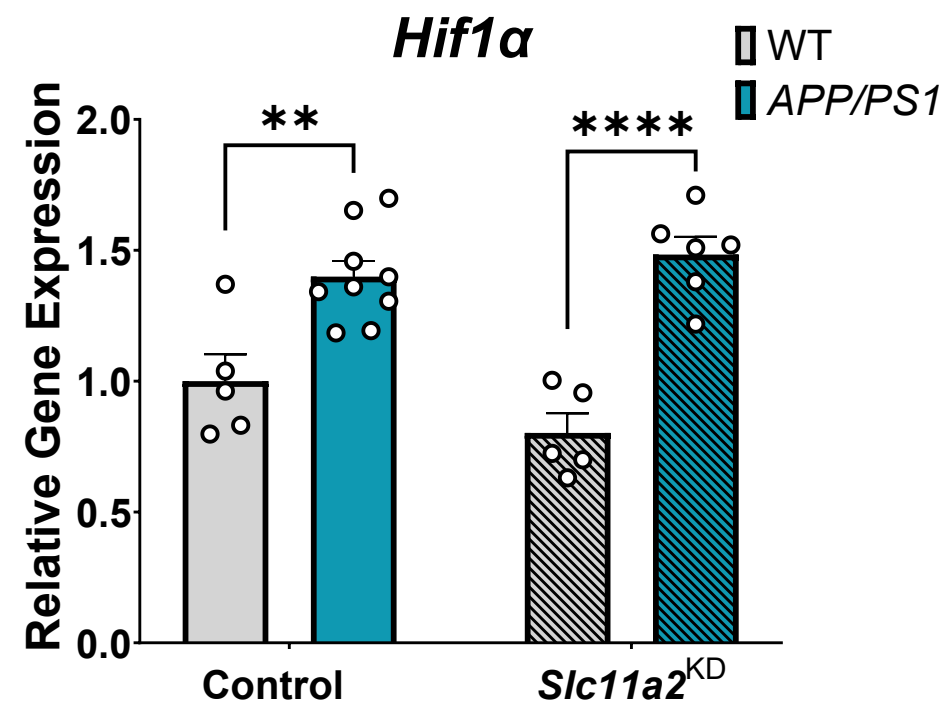**B**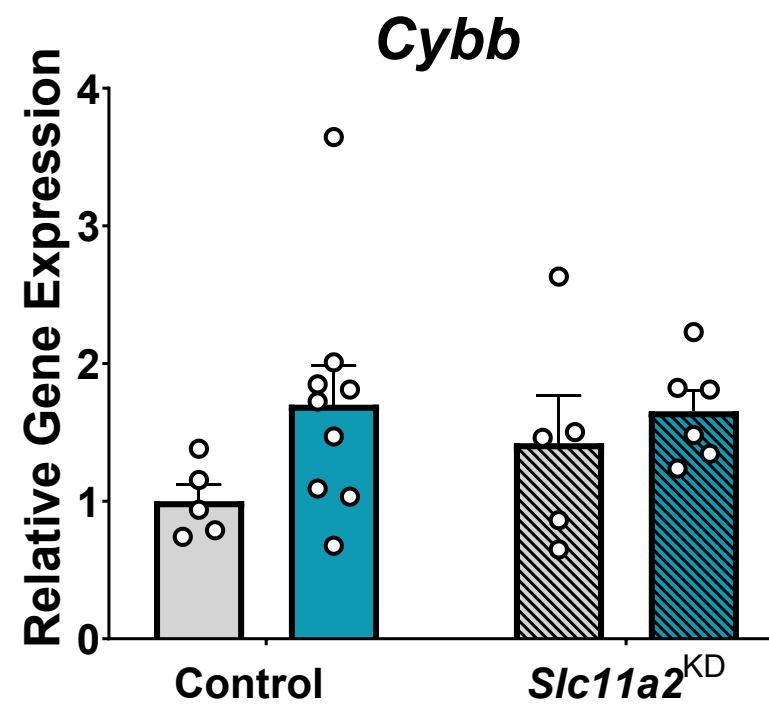**C**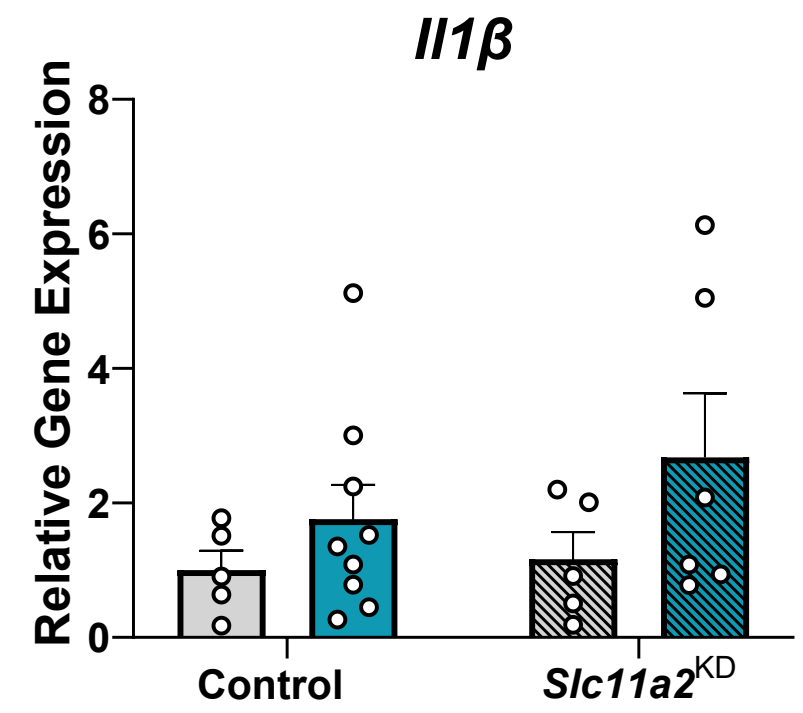

Supplement: Supplementary file 9 — Additional file 9. Slc11a2 knockdown had no significant effect on APP/PS1-associated increases in Hif1α, Cybb, or Il1β in male hippocampal microglia. A-C) RT-qPCR gene expression analysis of A Hif1α, B Cybb, and C Il1β from isolated hippocampal CD11b+ microglia from male mice. Two-way ANOVA, **p<0.01, ****p<0.0001. Data represent the mean ± S.E.M. of 5-9 mice per group. [file 12974_2024_3238_MOESM9_ESM.pdf]
