# Supplementary material for: Knockdown of microglial iron import gene, Slc11a2, worsens cognitive function and alters microglial transcriptional landscape in a sex-specific manner in the APP/PS1 model of Alzheimer’s disease
Source: J Neuroinflammation. 2024 Sep 27;21:238. doi: 10.1186/s12974-024-03238-w (PMC11438269; doi:10.1186/s12974-024-03238-w)
Supplement: Supplementary file 2 — Additional file 2. Mouse numbers and weights used in experiments. Supplemental Table 1 Mouse numbers used for behavioral assays. Supplemental Table 2 Body weights were assessed at time of euthanasia when mice were 15-18 months old. The data are presented as average weight in grams ± S.E.M. for 8–14 mice per group. One mouse from the male Slc11a2KD;APP/PS1 group and one female Control APP/PS1 mouse died prior to euthanasia. [file 12974_2024_3238_MOESM2_ESM.pdf]

**Supplemental Table 1**

| | |
|---|---|
| **Male Control** | 11 WT<br>14 *APP/PS1*+ |
| **Male *Slc11a2*$^{KD}$** | 12 WT<br>15 *APP/PS1*+ |
| **Female Control** | 12 WT<br>11 *APP/PS1*+ |
| **Female *Slc11a2*$^{KD}$** | 8 WT<br>13 *APP/PS1*+ |

**Supplemental Table 2**

| | **Control WT** | ***Slc11a2*$^{KD}$ WT** | **Control *APP/PS1*** | ***Slc11a2*$^{KD}$ *APP/PS1*** |
|---|---|---|---|---|
| Males | 37.49 ± 1.79 | 34.13 ± 0.99 | 37.06 ± 1.21 | 38.75 ± 1.18 |
| Females | 29.63 ± 1.32 | 29.89 ± 2.56 | 31.69 ± 0.95 | 29.95 ± 0.62 |
