# Supplementary material for: Knockdown of microglial iron import gene, Slc11a2, worsens cognitive function and alters microglial transcriptional landscape in a sex-specific manner in the APP/PS1 model of Alzheimer’s disease
Source: J Neuroinflammation. 2024 Sep 27;21:238. doi: 10.1186/s12974-024-03238-w (PMC11438269; doi:10.1186/s12974-024-03238-w)
Supplement: Supplementary file 3 — Additional file 3. Antibodies and primers used. Supplemental Table List of antibodies used in immunofluorescent staining of isolated glia, and list of gene primers used for RT-qPCR [file 12974_2024_3238_MOESM3_ESM.pdf]

Supplemental Table 3.

**Immunofluorescence**			
**Fluorophore/Antibody**	**Catalog number**	**Company**	**Dilution**
F4/80	ab16911	Abcam	1:200
Ferritin-L	ab69090	Abcam	1:500
DAPI	62248	ThermoFisher	1:4000
Goat anti-Rabbit IgG cross-adsorbed secondary antibody, Alexa Fluor 488	A-11008	ThermoFisher	1:1000
Goat anti-Rat IgG H&L, secondary antibody, Alexa Fluor 555	ab150158	Abcam	1:1000
**Gene Primers**			
**Gene Target**	**Catalog number**	**Company**	**Assay ID**
ActB	4351370	ThermoFisher	Mm00607939_s1
Cybb	4331182	ThermoFisher	Mm00432777_m1
Egr1	4331182	ThermoFisher	Mm00656724_m1
Ftl1	4331182	ThermoFisher	Mm03030144_g1
Fth1	4331182	ThermoFisher	Mm00850707_g1
Gapdh	4351370	ThermoFisher	Mm99999915_g1
Hif1α	4331182	ThermoFisher	Mm00468869_m1
Il1β	4331182	ThermoFisher	Mm00434228_m1
Il6	4351370	ThermoFisher	Mm00446190_m1
Mrc1	4331182	ThermoFisher	Mm0048148_m1
Nos2	4331182	ThermoFisher	Mm00440485_m1
Slc11a2, exons 15-16	4331182	ThermoFisher	Mm00435363_m1
Slc11a2, exons 7-8	4351372	ThermoFisher	Mm00435356_g1
Slc40a1	4331182	ThermoFisher	Mm01254822_m1
Tfrc	4331182	ThermoFisher	Mm00441941_m1
Tnfα	4331182	ThermoFisher	Mm00443258_m1
18S	4333760F	ThermoFisher	Hs99999901_s1
